# Supplementary material for: Mitochondrial phosphatase PGAM5 modulates cellular senescence by regulating mitochondrial dynamics
Source: Nat Commun. 2020 May 21;11:2549. doi: 10.1038/s41467-020-16312-7 (PMC7242393; doi:10.1038/s41467-020-16312-7)
Supplement: Supplementary file 1 — Supplementary Information [file 41467_2020_16312_MOESM1_ESM.pdf]

Supplementary Information

Mitochondrial Phosphatase PGAM5 modulates cellular senescence by regulating mitochondrial dynamics

Yu et al.

Supplementary Fig. 1

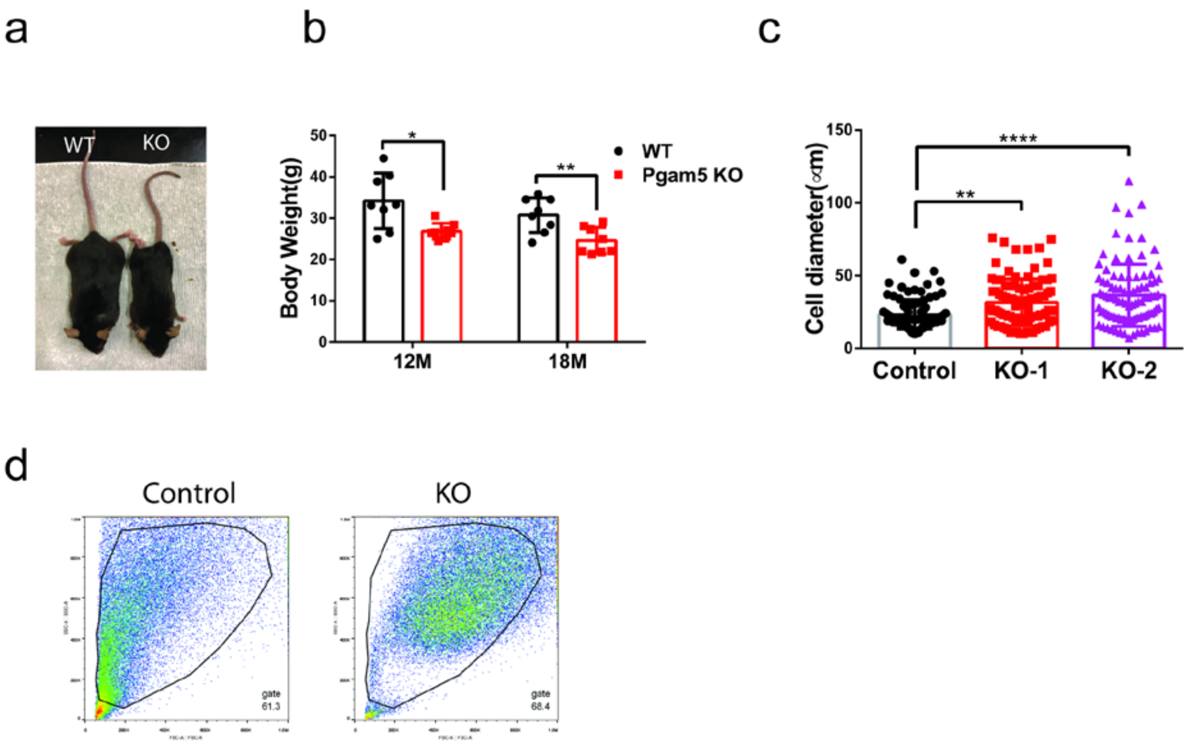

Supplementary Figure 1: Relative to Fig 1 and Fig 2. Reduced body weight in *Pgam5*$^{-/-}$ mice and increased cell size in *PGAM5*$^{-/-}$ cells.

(a) Gross image of WT and *Pgam5*$^{-/-}$ mice at 18 months-old.

(b) Reduced body weight in *Pgam5*$^{-/-}$ mice at 12 and 18 months old. n=8 biologically independent animals, *: p= 0.0102, **: p=0.005, two-tailed unpaired *t* tests, Error bars, mean ± s.d.;

(c) Cell size quantification through phase contrast images. n=95cells counted. **: p<0.005, ****: p<0.0001, one-way ANOVA Dunnett's multiple comparisons test. Error bars, mean ± s.d..

(d) Gating strategy was provided as required by Reporting Summary to Fig.2C. n=3 biologically independent experiments with similar results, representative figures were shown;

Source data are available as a Source Data file.

Supplementary Fig. 2

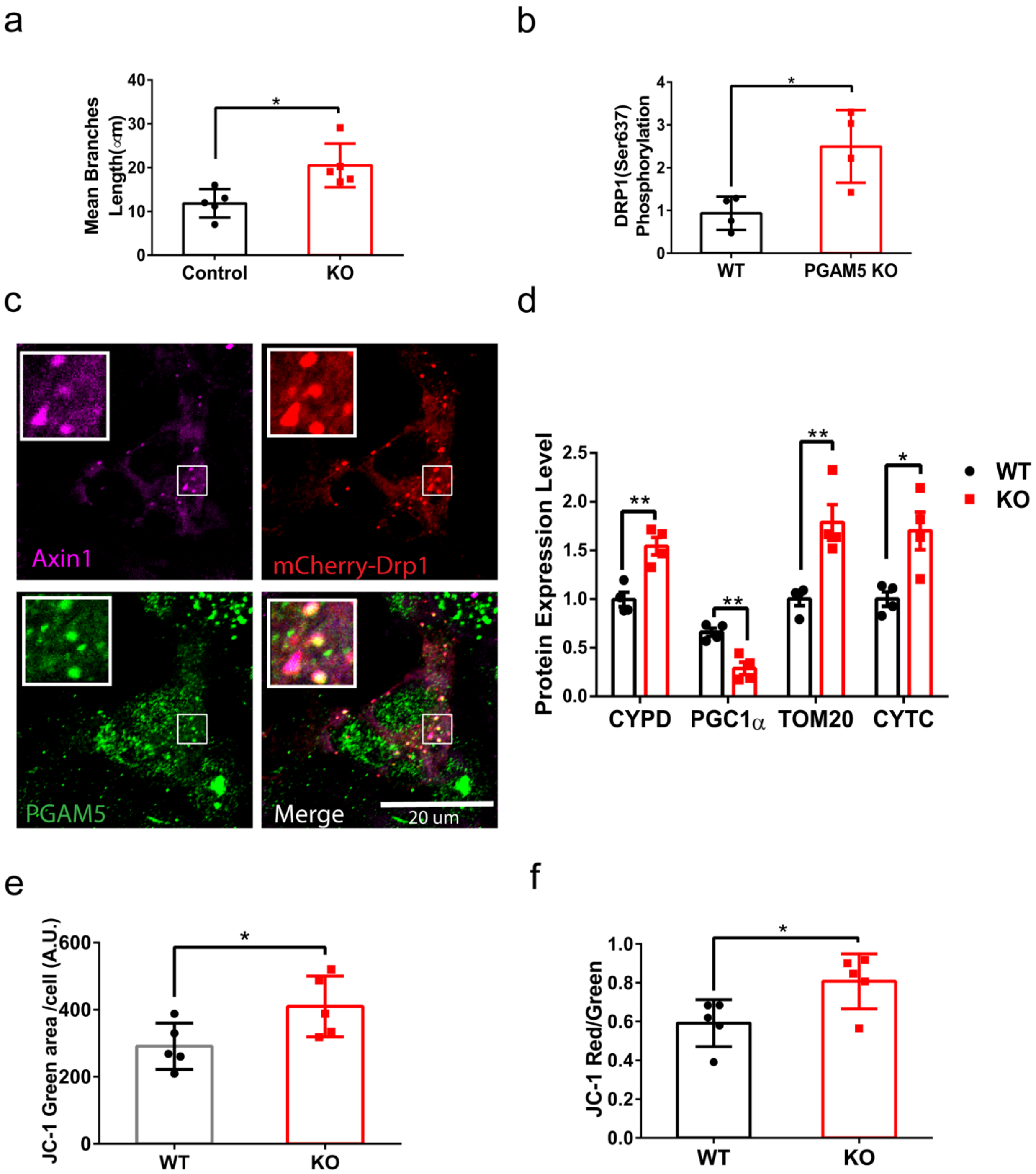

Supplementary Fig. 2: Relative to Fig.3. Mitochondrial feature changes in *PGAM5*$^{-/-}$ cells.

(a) Quantification of mitochondrial branch length in Fig. 3a. n=5 biologically independent experiments, *: p=0.0116. two-tailed unpaired *t* tests, Error bars, mean ± s.d.;

(b) Quantification of phosphor-Drp1(Ser637) increase in *PGAM5*$^{-/-}$ ARPE-19 cells in Fig.3b. n=4 biologically independent experiments, *: p=0.0155. two-tailed unpaired *t* tests, Error bars, mean ± s.d.;

(c) mCherry-Drp1(Addgene, #49152), Axin1 and PGAM5 co-localization in ARPE-19 cells. Scale Bar equals to 20um. n= 3 biologically independent experiments with similar results, representative images were shown;

(d) Quantification of mitochondrial protein expression in WT and *PGAM5*$^{-/-}$ ARPE-19 cells in Fig 3e. n=4 biologically independent experiments, *: p=0.0154 (CYTC), **: p=0.0032 (CYPD), p=0.0027 (PGC1α), p=0.007 (TOM20). two-tailed unpaired *t* tests, Error bars, mean ± s.d.;

(e) Quantification of JC-1 green signal in WT and *PGAM5*$^{-/-}$ ARPE-19 cells in Fig. 3i. n=5 biologically independent experiments, *: p=0.0481. two-tailed unpaired *t* tests, Error bars, mean ± s.d.;

(f) Quantification of red/green signal ratio of JC-1 in WT and *PGAM5*$^{-/-}$ ARPE-19 cells in Fig. 3i. n=5 biologically independent experiments, *: p=0.0323. two-tailed unpaired *t* tests, Error bars, mean ± s.d..

Source data are available as a Source Data file.

Supplementary Fig. 3

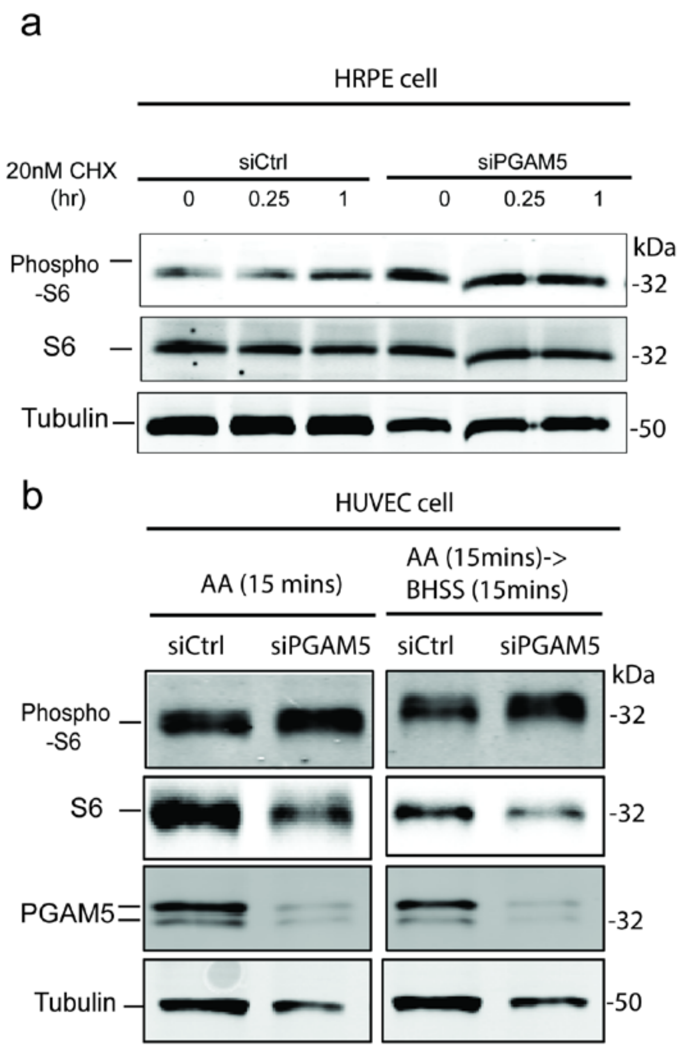

Supplementary Fig. 3: Relative to Fig.4. Enhancement of mTORC1 pathway by PGAM5 silencing in primary HRPE and HUVEC cells.

(A) Primary human RPE cells were transfected with control and PGAM5 siRNAs for 48 hours respectively. Western blot showed increased phospho-S6 but not S6 in PGAM5-silenced HRPE cells after treatment with 20mM CHX for indicated times. α-Tubulin was used as loading control.

n= 3 biologically independent experiments with similar results, representative images were shown;

(B) HUVEC cells were transfected with control and PGAM5 siRNAs for 48 hours respectively. After overnight serum starvation overnight, 1× Amino acid mix (Gibico, 11130051) was added into medium for 15 minutes. Western blot showed increased phosphor-S6 but not S6 in PGAM5-silenced HUVEC cells after treatment (left panel). In the right panel, medium was replaced by HBSS for another 15 minutes of incubation before cell harvest. α-Tubulin was used as loading control. n= 3 biologically independent experiments with similar results, representative images were shown.

Source data are available as a Source Data file.

Supplementary Fig. 4

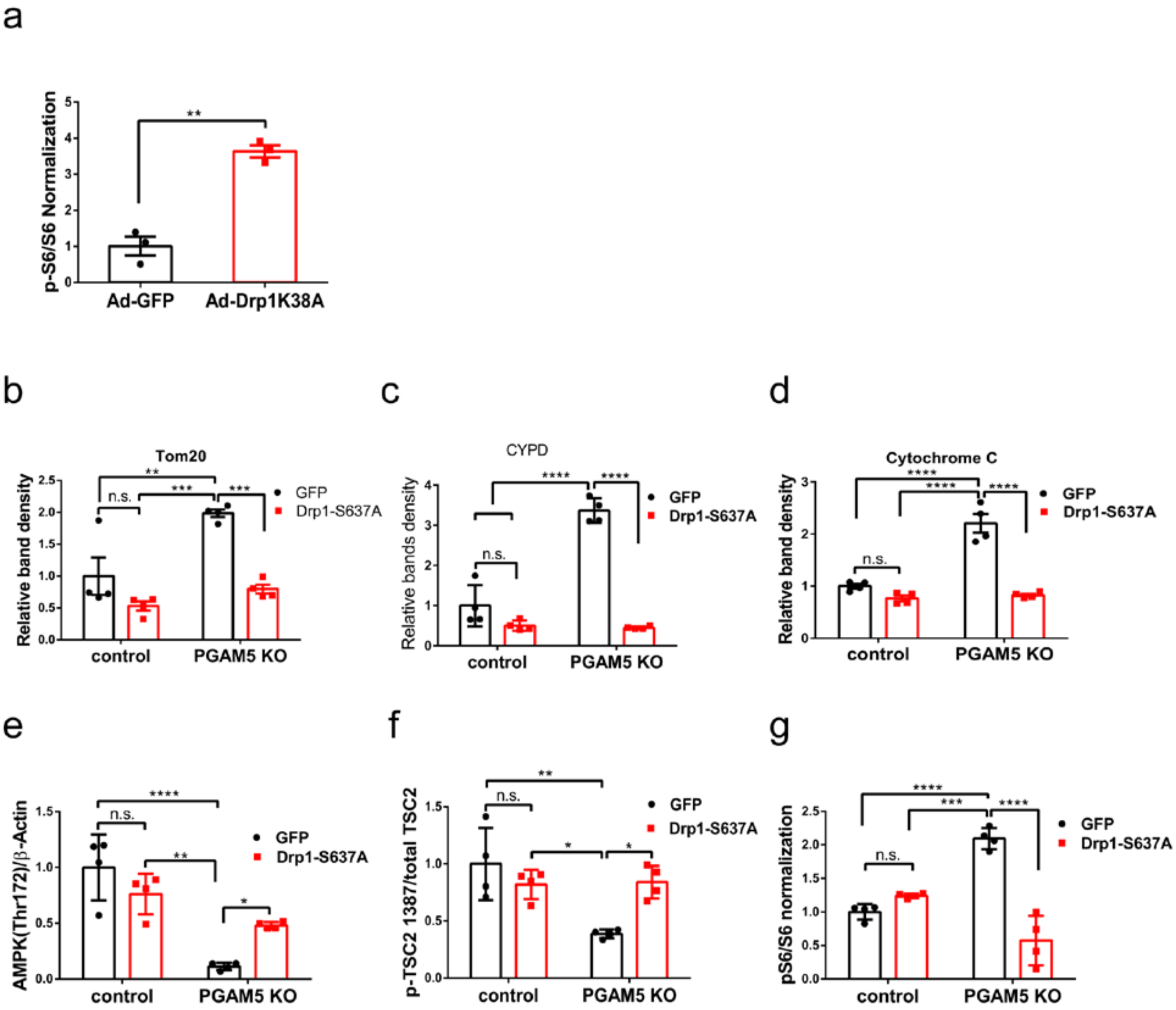

Supplementary Fig.4: Relative to Fig.5 and Fig.6. Drp1K38A mimics and Drp1S637A rescues AMPK-mTORC1 pathway in *PGAM5*$^{-/-}$ cells.

(a) Quantification of phosphor-S6 in ARPE-19 cells overexpressing Ad-LacZ or Ad-Drp1K38A for 7 days in Fig.5e. n=3 biologically independent experiments, **: p=0.0011. two-tailed unpaired *t* tests, Error bars, mean ± s.d.;

(b) Quantification of Tom20 in APRE-19 control or *PGAM5*$^{-/-}$ cells overexpressing lenti-GFP or lenti-Drp1 S637A for one week in Fig 6c. n=4 biologically independent experiments. **: p<0.001, ***: p<0.0005, n.s.: no significant, two-way ANOVA Tukey's multiple comparisons test. Error bars, mean ± s.d.;

(c) Quantification of CYPD in APRE-19 control or *PGAM5*$^{-/-}$ cells overexpressing lenti-GFP or lenti-Drp1 S637A for one week in Fig 6c. n=4 biologically independent experiments. ****: p<0.0001, n.s.: no significant, two-way ANOVA Tukey's multiple comparisons test. Error bars, mean ± s.d.;

(d) Quantification of Cytochrome C in APRE-19 control or *PGAM5*$^{-/-}$ cells overexpressing lenti-GFP or lenti-Drp1 S637A for one week in Fig6c. n=4 biologically independent experiments. ****: p<0.0001, n.s.: no significant, two-way ANOVA Tukey's multiple comparisons test. Error bars, mean ± s.d.;

(e) Quantification of phosphor-AMPK(Thr-172) level in APRE-19 control or *PGAM5*$^{-/-}$ cells overexpressing lenti-GFP or lenti-Drp1 S637A for one week in Fig 6d. n=4 biologically independent experiments. *: p<0.05, **: p<0.01, ****: p<0.0001, n.s.: no significant, two-way ANOVA Tukey's multiple comparisons test. Error bars, mean ± s.d.;

(f) Quantification of phosphor-TSC2 1387 level in APRE-19 control or *PGAM5*$^{-/-}$ cells overexpressing lenti-GFP or lenti-Drp1 S637A for one week in Fig 6d. n=4 biologically independent experiments. *: p<0.05, **: p<0.01, n.s.: no significant, two-way ANOVA Tukey's multiple comparisons test. Error bars, mean ± s.d.;

(g) Quantification of phosphor-S6 level in APRE-19 control or *PGAM5*$^{-/-}$ cells overexpressing lenti-GFP or lenti-Drp1 S637A for one week in Fig 6d. n=4 biologically independent

experiments. ***: p<0.001, ****: p<0.0001, n.s.: no significant, two-way ANOVA Tukey's multiple comparisons test. Error bars, mean ± s.d..

Source data are available as a Source Data file.

Supplementary Fig.5

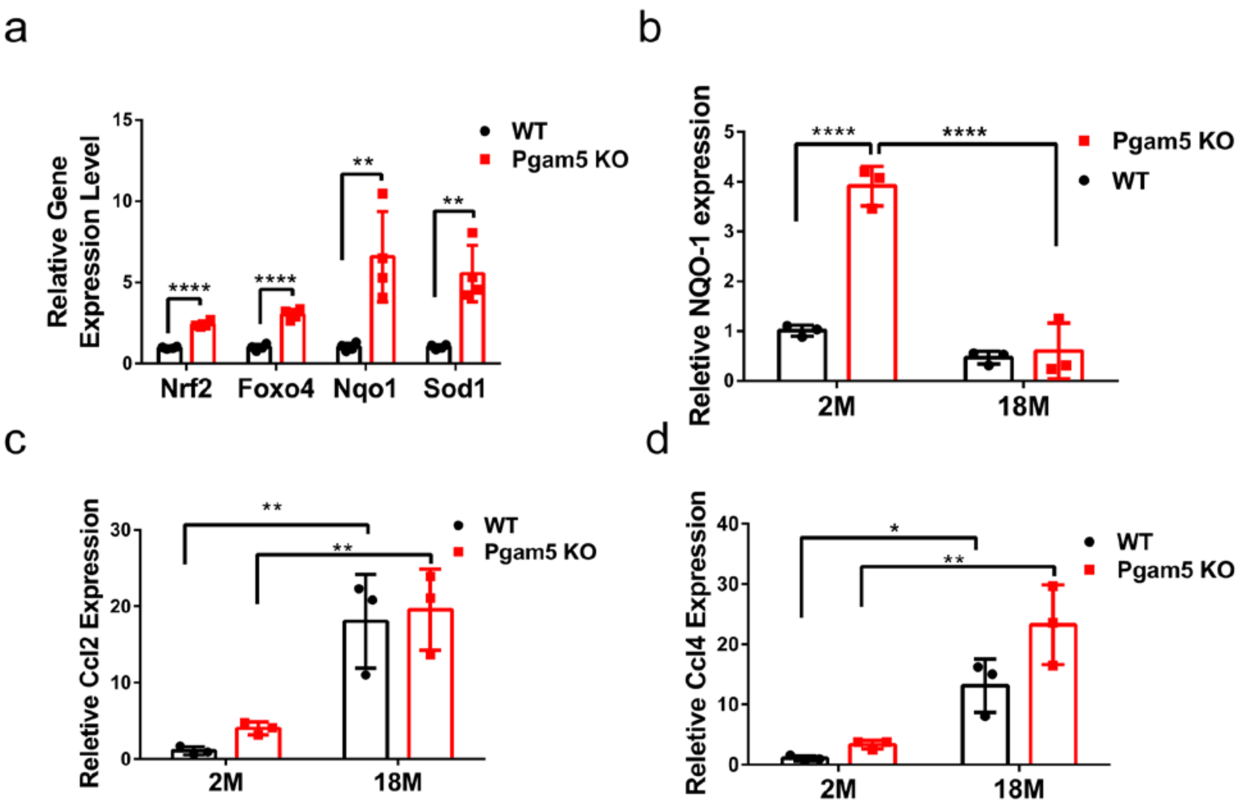

Supplementary Fig. 5: Relative to Fig.7, Fig.8 and Fig.9. Upregulation of antioxidant and inflammatory genes in *Pgam5*$^{-/-}$ mice.

(a) Upregulation of anti-oxidative genes (Nrf2, FoxO4, Sod1, Nqo1) in 2-months-old RPE/choroid of *Pgam5*$^{-/-}$ mice compared to the control mice by qRT-PCR. n=4 biologically independent experiments, **: p=0.0073 (NQO1), p=0.0021 (SOD1), ****: p<0.0001(Nrf2 and FoxO4). two-tailed unpaired *t* tests, Error bars, mean ± s.d.;

(b) *Nqo-1* expression in RPE/choroid of 2 months or 18 months-old WT and *Pgam5*$^{-/-}$ mice. n=3 biologically independent experiments, ****: p<0.0001. two-way ANOVA Tukey's multiple comparisons test. Error bars, mean ± s.d.;

(c) Upregulation of *Ccl2* in 2- and 18-months-old RPE/choroid of *Pgam5*$^{-/-}$ mice compared to the control mice by qRT-PCR. n=3 biologically independent animals in each group, **: p<0.005. two-way ANOVA Tukey's multiple comparisons test. Error bars, mean ± s.d.;

(d) Upregulation of *Ccl4* in 2- and 18-months-old RPE/choroid of *Pgam5*$^{-/-}$ mice compared to the control mice by qRT-PCR. n=3 biologically independent animals in each group, *: p<0.05, **: p<0.005, two-way ANOVA Tukey's multiple comparisons test. Error bars, mean ± s.d..

Source data are available as a Source Data file.

Supplementary Fig.6

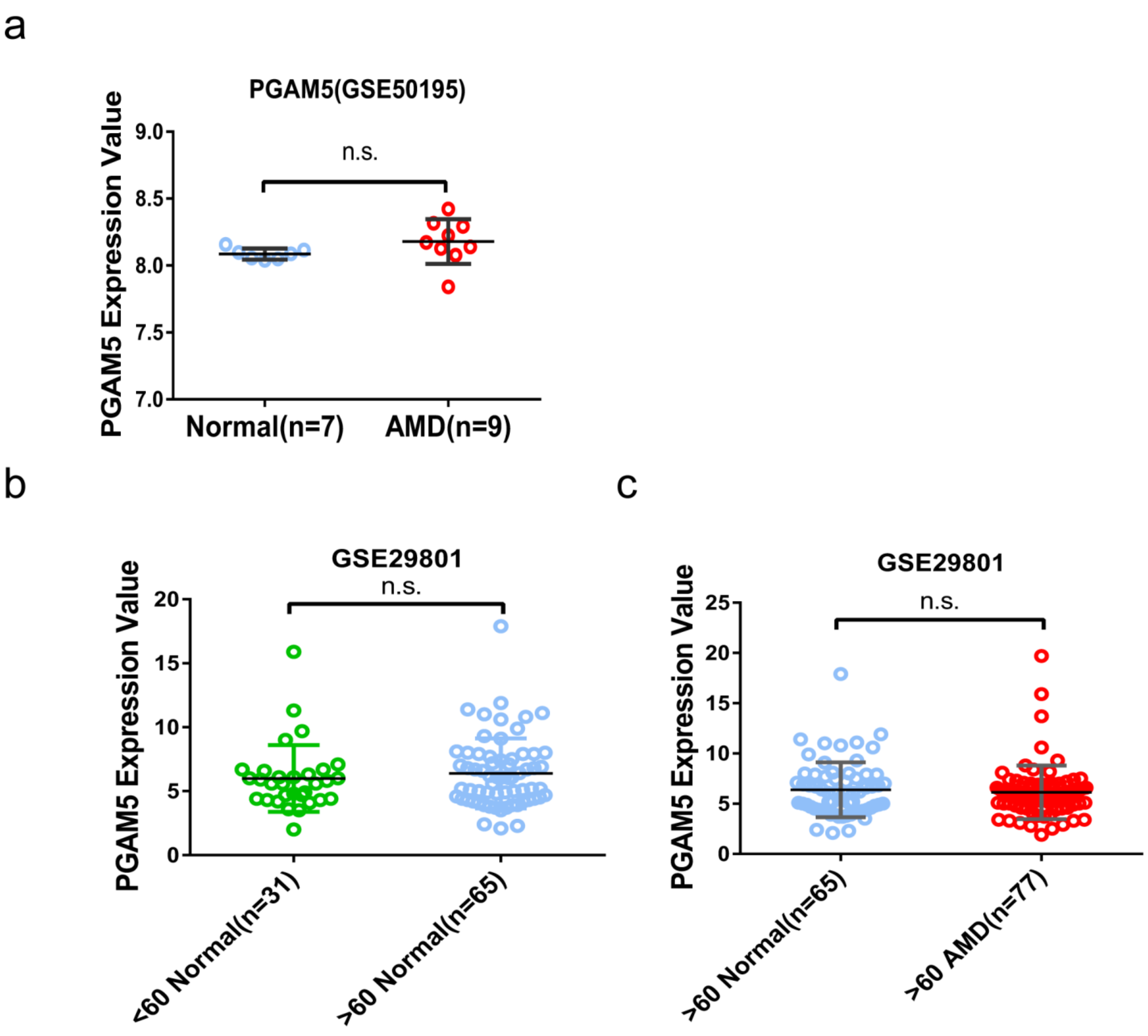

Supplementary Fig. 6: Relative to Discussion Section. *PGAM5* mRNA expression is not altered with age or in AMD disease.

(A) *PGAM5* mRNA expression in healthy and AMD people. Data from GSE50195. n.s.: no significant. two-tailed unpaired *t* tests, Error bars, mean ± s.d.;

(B) *PGAM5* mRNA expression in <60 and >60 years of old people. Data from GSE29801. n.s.: no significant. two-tailed unpaired *t* tests, Error bars, mean ± s.d.;

(C) *PGAM5* mRNA expression in healthy (>60 years) and AMD (>60 years) people. Data from GSE29801. n.s.: no significant. two-tailed unpaired *t* tests, Error bars, mean ± s.d.;

Source data are available as a Source Data file.

Supplementary Table 1

Primers used in the manuscript

qhNrf2-F:	AGGTTGCCCACATTCCCAAA
qhNrf2-R:	ACGTAGCCGAAGAAACCTCA
qhSOD1-F:	ACAAAGATGGTGTGGCCGAT
qhSOD1-R:	AACGACTTCCAGCGTTTCCT
qhFOXO4-F:	CCGACCAGAGATCGCTAACC
qhFOXO4-R:	TGATTTCCCCAGGCATTCCG
qmNrf2-F:	AAGAATAAAGTCGCCGCCCA
qmNrf2-R:	AGATACAAGGTGCTGAGCCG
qhIFNb1-F:	TCTCCTGTTGTGCTTCTCCAC
qhIFNb1-R:	GCCTCCCATTCAATTGCCAC
qmIFNb-F:	AAGAGTTACACTGCCTTTGCCATC
qmIFNb-R:	CACTGTCTGCTGGTGGAGTTCATC
qhTNFa-F:	TGGGATCATTGCCCTGTGAG
qhTNFa-R:	GGTGTCTGAAGGAGGGGGTA
qhMMP3-F:	AAAGACAGGCACTTTTGGCG
qhMMP3-R:	CTTCATATGCGGCATCCACG
qhIL6-F:	CTCAATATTAGAGTCTCAACCCCCA
qhIL6-R:	GAGAAGGCAACTGGACCGAA
qmTNFa-F:	TGCTCTGTGAAGGGAATGGG
qmTNFa-R:	ACCCTGAGCCATAATCCCCT
qmIL6-F:	CCCCAATTTCCAATGCTCTCC
qmIL6-R:	CGCACTAGGTTTGCCGAGTA
qmMMP3-F:	AAGTTGTCAAAGGATGTTCAGAAG
qmMMP3-R:	ACAGTTCAGACACTTGTTTGTGG
qmCCL2-F:	CCCCAAGAAGGAATGGGTCC
qmCCL2-R:	GTGCTGAAGACCTTAGGGCA
qmCCL4-F:	CGTTCAGATTTCCTGCCCCT
qmCCL4-R:	CATCTCCATGGGAGACACGC
qmNQO1-F:	AGCCAATCAGCGTTCGGTAT
qmNQQ1-R:	GCCTCCTTCATGGCGTAGTT
qmSOD1-F:	ATTGGCCGTACAATGGTGGT
qmSOD1-R:	ATCCCAATCACTCCACAGGC
qmFOXO4-F:	GCTAGGCGGAGGTTACAGG
qmFOXO4-R:	GAACTTCTCCCTCGTCTCCG
qhbeta-actin-F:	CACCAACTGGGACGACAT
qhbeta-actin-R:	ACAGCCTGGATAGCAACG
qhGAPDH-F:	GGATTTGGTCGTATTGGG
qhGAPDH-R:	GGAAGATGGTGATGGGATT
qmbeta-actin-F:	CTGTCCCTGTATGCCTCTG

qmbeta-actin-R:	ATGTCACGCACGATTTCC
qmGAPDH-F:	GTTGTCTCCTGCGACTTCA
qmGAPDH-R:	GGTGGTCCAGGGTTTCTTA
PGAM5 KO-1-F (sgRNA)	caccgTCTTCTCGGCCGTGGCGGTA
PGAM5 KO-1-R (sgRNA)	aaacTACCGCCACGGCCGAGAAGAc
PGAM5 KO-2-F (sgRNA)	caccgGCGCGGCTTCCCTACCGCCA
PGAM5 KO-2-R (sgRNA)	aaacTGGCGGTAGGGAAGCCGCGCc

Supplementary Video 1

Representative behavior of WT and *Pgam5*$^{-/-}$ mice at 18 months. The mouse with ear ring is *Pgam5*$^{-/-}$ mouse, and the other one is WT. KO mouse shows Parkinson-like movements.
