# Supplementary material for: Mitochondrial phosphatase PGAM5 modulates cellular senescence by regulating mitochondrial dynamics
Source: Nat Commun. 2020 May 21;11:2549. doi: 10.1038/s41467-020-16312-7 (PMC7242393; doi:10.1038/s41467-020-16312-7)
Supplement: Supplementary file 4 — Reporting Summary [file 41467_2020_16312_MOESM4_ESM.pdf]

nature research

Corresponding author(s): Shusheng Wang

Last updated by author(s): Apr 12, 2020

Reporting Summary

Nature Research wishes to improve the reproducibility of the work that we publish. This form provides structure for consistency and transparency in reporting. For further information on Nature Research policies, see Authors & Referees and the Editorial Policy Checklist.

Statistics

For all statistical analyses, confirm that the following items are present in the figure legend, table legend, main text, or Methods section.

n/a	Confirmed	
☐	☒	The exact sample size (n) for each experimental group/condition, given as a discrete number and unit of measurement
☐	☒	A statement on whether measurements were taken from distinct samples or whether the same sample was measured repeatedly
☐	☒	The statistical test(s) used AND whether they are one- or two-sided<br>*Only common tests should be described solely by name; describe more complex techniques in the Methods section.*
☐	☒	A description of all covariates tested
☒	☐	A description of any assumptions or corrections, such as tests of normality and adjustment for multiple comparisons
☐	☒	A full description of the statistical parameters including central tendency (e.g. means) or other basic estimates (e.g. regression coefficient) AND variation (e.g. standard deviation) or associated estimates of uncertainty (e.g. confidence intervals)
☐	☒	For null hypothesis testing, the test statistic (e.g. F, t, r) with confidence intervals, effect sizes, degrees of freedom and P value noted<br>*Give P values as exact values whenever suitable.*
☒	☐	For Bayesian analysis, information on the choice of priors and Markov chain Monte Carlo settings
☒	☐	For hierarchical and complex designs, identification of the appropriate level for tests and full reporting of outcomes
☒	☐	Estimates of effect sizes (e.g. Cohen's d, Pearson's r), indicating how they were calculated

Our web collection on statistics for biologists contains articles on many of the points above.

Software and code

Policy information about availability of computer code

Data collection	Q-PCR was performed using the CFX96 Real Time PCR (Bio-rad) and Bio-Rad CFX Manager (version 3.1)<br>For flow cytometry, data were collected on Sony cell sorter and and analyzed by FlowJo V10.<br>Immunohistochemical staining images were collected by Ti-S microscopy and NIS-Elements(version BR 4.20.03 64 bit) from Nikon.<br>Immunofluorescence staining images were collected by A1 confocal microscopy(Nikon) and NIS-Elements AR Analysis Software(version 5.20.01 64-bit)
Data analysis	GraphPad Prism 6 Software was used for statistical analysis.<br>NIS-Elements Software (version 5.02.00 64 bit) was used for analysing the IHC staining and immunofluorescence staining images<br>Image J Software was used for analysing the IHC staining and immunofluorescence staining images.

For manuscripts utilizing custom algorithms or software that are central to the research but not yet described in published literature, software must be made available to editors/reviewers. We strongly encourage code deposition in a community repository (e.g. GitHub). See the Nature Research guidelines for submitting code & software for further information.

Data

Policy information about availability of data

All manuscripts must include a data availability statement. This statement should provide the following information, where applicable:

- Accession codes, unique identifiers, or web links for publicly available datasets
- A list of figures that have associated raw data
- A description of any restrictions on data availability

The data supporting the results of this study are available within the paper and its supplementary information files.
There is no restriction on material availability. We provide a statement on data availability in our manuscript, as required by Nature Policy.

Field-specific reporting

Please select the one below that is the best fit for your research. If you are not sure, read the appropriate sections before making your selection.

☒ Life sciences ☐ Behavioural & social sciences ☐ Ecological, evolutionary & environmental sciences

For a reference copy of the document with all sections, see nature.com/documents/nr-reporting-summary-flat.pdf

Life sciences study design

All studies must disclose on these points even when the disclosure is negative.

Sample size	To perform statistics and calculate the s.d. or s.e.m., at least three biologically independent experiments were performed. The exact sample size is indicated in each experiment.
Data exclusions	No data were excluded.
Replication	All relevant experiments were repeated for three times or above,unless stated otherwise. All repeats were also successful.
Randomization	Animals in the study were randomly assigned to experimental or control groups.
Blinding	Researchers were blinded to sample identity during image collection and data analysis.

Reporting for specific materials, systems and methods

We require information from authors about some types of materials, experimental systems and methods used in many studies. Here, indicate whether each material, system or method listed is relevant to your study. If you are not sure if a list item applies to your research, read the appropriate section before selecting a response.

Materials & experimental systems

n/a	Involved in the study	
☐	☒	Antibodies
☐	☒	Eukaryotic cell lines
☒	☐	Palaeontology
☐	☒	Animals and other organisms
☒	☐	Human research participants
☒	☐	Clinical data

Methods

n/a	Involved in the study	
☒	☐	ChIP-seq
☐	☒	Flow cytometry
☒	☐	MRI-based neuroimaging

Antibodies

Antibodies used	anti-Tom20 (1:1000,BD, 612278), anti-b-tubulin (1:1000.Abcam, ab6046) anti- P16Ink4a (1:1000,BD Biosciences, 550834), anti-Cytochrome C (1:1000, Abcam, ab110325), anti-phospho-IRF-3 (Ser396) (1:1000, Cell Signaling Technologist, 4947) anti-CYPD (1:1000. Abcam, ab110324), anti-a-tubulin (Millipore, 05-829), anti-b-actin (1:2000, Santa Cruz, sc-47778), anti-PGAM5 (1:1000, Santa Cruz, A-3) ,anti-p-Drp1637 (1:1000, Abcam, ab193216), anti-pS6 (1:1000 for WB, 1:400 for IF, Cell Signaling, 2211), anti-S6 (1:1000, Cell Signaling, 2317), anti-phosphor-AMPK(172) (1:1000, Cell Signaling, 2535), anti-total-AMPK(1:1000. Cell Signaling, 2532), anti-phosphor-TSC2(1387) (1:1000, Cell Signaling, 5584T), anti-Lamin B1 (1:1000, Santa Cruz, sc-377000), anti-MMP3 (1:1000, Chemicon International, MAB1339),anti-macroH2A (1:1000, Abcam, ab37264) anti-phosphor-4EBP1(37/46) (1:1000, Cell Signaling, 28555), anti-Axin1(1:1000, Cell Signaling, 2087S) ,anti-Nrf2(1:1000, Santa Cruz,sc-365949) anti-Drp1 total (1:1000, Abcam, ab56788), anti-gamma H2A.X (phospho S139)(1:400, Abcam,ab81299), anti-Drp1 total(1:1000,Santa Cruz,sc-271583),phoillodin-488(1:400, Cell Signaling, 8878), anti-Zo1(1:400, invitrogen, MA3-39100-A647), anti-IBA1(1:400, FUJIFILM, 019-19741)anti- P16Ink4a(1:400, Abcam,ab54210), goat anti-Mouse 800 secondary antibody(1:3000, Licor, 926-32210), goat anti-Rabbit 680 secondary antibody(1:3000, Licor,926-68071)
Validation	1.anti-Tom20 (BD, 612278) was verified in Abe Y, Shodai T, Muto T, et al.Cell. 2000; 100(5):551-560.<br>2.anti-b-tubulin (Abcam, ab6046) was verified in de Sousa FD et al. Int J Biol Macromol 121:429-442 (2019).<br>3.anti- P16Ink4a (BD Biosciences, 550834)was verified in de Sousa FD et al. Int J Biol Macromol 121:429-442 (2019).<br>4.anti-Cytochrome C (Abcam, ab110325) was verified in Zhao Y et al. Nat Commun 10:1371 (2019).<br>5.anti-phospho-IRF-3 (Ser396) (Cell Signaling Technologist, 4947) was verified in Tea Carletti et al. Nat Commu 10:3889(2019)<br>6.anti-CYPD (Abcam, ab110324) was verified in Gräb J et al Nat Commun 10:688 (2019).<br>7.anti-!-tubulin (Millipore, 05-829) was verified in Choi HK et al. Nature communications 6 7390 (2015).<br>8.anti-b-actin (Santa Cruz, sc-47778) was verified in Davis, MA et al. Proc. Natl. Acad. Sci. U.S.A. 116(11):5061-5070.(2019)<br>9. anti-PGAM5 (Santa Cruz, A-3) was verified by anti-PGAM5(abcam,126534), which was verified by Bernkopf DB et al J Cell Biol 217:1383-1394 (2018).<br>10.anti-p-Drp1637 (Abcam, ab193216) was verified in Gong Y et al Aging (Albany NY) 10:3104-3116 (2018).<br>11. anti-pS6 (Cell Signaling, 2211) was verified in Diernfellner,A.C.R. et al. Proc. Natl. Acad. Sci. U.S.A.116(35):17271–17279.

(2019)
12.anti-S6 (Cell Signaling, 2317) was verified in Katayama, R., Gong, B., et al.Nat Commu 10:3604(2019).
13.anti-phosphor-AMPK(172) (Cell Signaling, 2535) was verified in Seoane-Collazo, P.et al. Nat Commu 10:4037(2019).
14.anti-total-AMPK(Cell Signaling, 2532) was verified in Inyang, K. E.et al.J Pharmacol Exp Ther 371:138–150(2019).
15.anti-phosphor-TSC2(1387) (Cell Signaling, 5584T) was verified in Lorena et.al. Autophagy, 15:9, 1572-1591(2019).
16.anti-Lamin B1 (Santa Cruz, sc-377000) was verified in Li H et al.Dev Cell. Apr 8;49(1):118-129.e7(2019).
17.anti-MMP3 (Chemicon International, MAB1339) was verified in Shahed A et al.Mol Reprod Dev. Sep;75(9):1433-40 (2008).
18.anti-macroH2A (Abcam, ab37264) was verified in Haque N et al.Nat Commun 9:1145 (2018).
19.anti-phosphor-4EBP1(37/46) (Cell Signaling, 28555) was verified in Schreiber, K. H.et al. Nat Commun 10:3194(2019).
20.anti-Axin1(Cell Signaling, 2087S) was verified in Ji, L.et al.Nat Commu Nat Commun 10:4184(2019).
22.anti-Nrf2(Santa Cruz,sc-365949) was verified in Shou JW et al. Front Cell Neurosci.(2019)
23.anti-Drp1 total (Abcam, ab56788) was verified inWard JM et al. Cell Rep 26:1189-1202.e6 (2019).
24.anti-Drp1 total (Santa Cruz,sc-271583) was verified in Shengchen Li et al. Cell Rep 2019 Sep10;28:2824-2836.e8 (2019).
25.phoillodin-488(*****)was verified in Shengchen Li et al. Cell Rep 2019 Sep10;28:2824-2836.e8 (2019).
26.anti- P16Ink4a (Abcam,ab54210) was verified in Montero-Melendez T et al. Nat Commun 11:745 (2020)..
27.anti-gamma H2A.X (phospho S139)(Abcam,ab81299) was verified in Mangeot PE et al.Nat Commun 10:45 (2019).
28.Zo-1 antibody(invitrogen, MA3-39100-A647) was verified in Pena-Philippides et al. J Am Heart Assoc. Jul 3; 7(13): (2018)
29.pholloidin-488(Cell Signaling, 8878) was verified in Sakurikar, N. et al. Oncotarget 7, 1380-94.(2016)
30. anti-IBA-1 antibody (FUJIFILM, 019-19741) was verified in Wan, S., et al.: J. Neuroinflammation, 15, 31(2018).

Eukaryotic cell lines

Policy information about cell lines

Cell line source(s)	ARPE19 cells from ATCC, HRPE cells and HUVEC cells from Lonza. AD-293 cells from Agilent. ES cells fromEuropean Mouse Mutant Cell Repository.
Authentication	None of the cell lines used were authenticated.
Mycoplasma contamination	All the cell lines were tested negative for mycoplasma contamination.
Commonly misidentified lines (See ICLAC register)	No commonly misidentified lines were used in this study.

Animals and other organisms

Policy information about studies involving animals; ARRIVE guidelines recommended for reporting animal research

Laboratory animals	C57BL/6J WT and PGAM5-/- mice were used in this study. The mouse age was indicated in the manuscript.
Wild animals	No wild animals were used in this study.
Field-collected samples	No field-collected samples were used in this study.
Ethics oversight	Animal research is monitored by the Institutional Animal Care and Use Committees(IACUCs) in Tulane University.

Note that full information on the approval of the study protocol must also be provided in the manuscript.

Flow Cytometry

Plots

Confirm that:

☒ The axis labels state the marker and fluorochrome used (e.g. CD4-FITC).

☒ The axis scales are clearly visible. Include numbers along axes only for bottom left plot of group (a 'group' is an analysis of identical markers).

☒ All plots are contour plots with outliers or pseudocolor plots.

☒ A numerical value for number of cells or percentage (with statistics) is provided.

Methodology

Sample preparation	Control and PGAM5 KO ARPE-19 cells were cultured for 8 weeks before being digested by trypsin from dishes. Cells were rinsed two times by PBS buffer and filtered through 45um cell strainer before being loaded to cell sorter.
Instrument	Sony SH800S
Software	Sony SH800S Cell Sorter Software(version 2.1.5)
Cell population abundance	23176 cells were counted for WT cells. 34393 cells were counted for PGAM5-/- cells.

Gating strategy	FSC is of interest target

☒ Tick this box to confirm that a figure exemplifying the gating strategy is provided in the Supplementary Information.
